# Comparison of EuroQol-5D-3L and Short Form-6D Utility Scores in Family Caregivers of Colorectal Cancer Patients: A Cross-Sectional Survey in China

**DOI:** 10.3389/fpubh.2021.742332

**Published:** 2021-09-29

**Authors:** Cheng-yao Sun, Yang Liu, Liang-ru Zhou, Ming-si Wang, Xian-ming Zhao, Wei-dong Huang, Guo-xiang Liu, Xin Zhang

**Affiliations:** ^1^Department of Health Economics, College of Health Management of Harbin Medical University, Harbin, China; ^2^Department of Health Education, College of Public Health of Harbin Medical University, Harbin, China; ^3^Tumor Radiotherapy Center, Harbin the First Hospital, Harbin, China

**Keywords:** family caregivers, colorectal cancer, EQ-5D, SF-6D, quality of life

## Abstract

**Objective:** To compare the EuroQol-5D-3L (EQ-5D-3L) and the Short Form-6D (SF-6D) utility scores in family caregivers (FCs) of colorectal cancer (CRC) patients.

**Method:** This study was performed on FCs of CRC patients from three primary cancer centers in the capital city of the Heilongjiang province. The participants (FCs) who were enrolled, filled the EQ-5D-3L, along with the SF-6D questionnaire. Two tools were compared for their distribution, discriminant validity, agreement, and convergent validity along with known-groups validity.

**Result:** Two hundred ninety-two FCs of CRC patients were enrolled. The score distribution of the SF-6D along with the EQ-5D-3L were not normal. A ceiling impact was seen in 31.8% of the FCs for EQ-5D-3L; however, none for the SF-6D. Good associations (Spearman’s rho = 0.622, *p* < 0.01) and intraclass correlation coefficient (ICC 0.637 and average ICC 0.778) between the two scores were observed. The EQ-5D-3L yielded higher utility scores in contrast with the SF-6D in the better health subclass. The SF-6D distinguished better between excellent and good health statuses, with better effect size and relative efficiency statistics. Both tools showed good known-groups validity.

**Conclusion:** The utility scores of SF-6D were remarkably lower relative to that of the EQ-5D-3L, but the difference may be clinically insignificant. However, the SF-6D may be superior because of the lack of ceiling impact. SF-6D exhibited a better convergent validity along with discrimination validity of excellent health condition and improved known-groups validity efficiency.

## Introduction

The global prevalence and deaths due to colorectal cancer (CRC) have been on the rise (1). In China, CRC ranks third among the top leading types of cancer. There were ~376,300 new cases and ~191,000 fatalities from CRC in 2015 (2).

Caring for an individual with CRC can take a drastic toll on the physical as well as mental health of the family caregivers (FCs) (3, 4). Some hitherto research studies have adopted generic tools, for instance the SF-36 Health Survey Instrument, for evaluating the health-related quality of life (HRQoL) of the FCs (5). Although these investigations documented that FCs of individuals with cancer have remarkably worst psychological and social influences in contrast with others, they cannot be transformed into a single health utility score. A single utility score is used to depict the general public preference. Cost-utility assessment has been extensively adopted as an excellent approach for allocation of resources (6, 7). Indirect HRQoL determinants, for instance the Short Form-6D (SF-6D) along with the EuroQol-5D-3L (EQ-5D-3L), are usually employed to derive health condition values for computing QALY. Both instruments employ a distinct descriptive or classification approach to categorize diverse health conditions (8, 9). A health utility score of 1 indicates a condition of perfect health, whilst a utility score of 0 indicates being dead.

Hitherto investigators have compared these two tools and have indicated some discrepancies in their performances (10, 11). The EQ-5D-3L is extensively utilized and easily administered, but the performance of EQ-5D-3L in assessing small alterations in high-level utilities is relatively poor (12). Some investigations documented that SF-6D overestimated at the lower utility value levels and exhibited low responsivity to transform within a lower utility values range (8, 13). The difference between the two utility scores has not been demonstrated in the FCs of CRC patients. Herein, we proposed to compare the two tools regarding their distribution, known-groups validity, agreement, convergent validity, as well as discriminant validity.

## Patients and Methods

### Data Source and Collection

We carried out a cross-sectional survey assessment in Heilongjiang with a population of ~37.5 million. From December 2016 to April 2017, we recruited FCs of the CRC patients who were treated at three cancer centers located in Harbin, the capital city of the Heilongjiang province. These three hospitals were chosen as they offer specialist care to individuals with CRC over the whole province. FCs of CRC patients need to meet the following criteria: First, the CRC patients had to have a confirmed diagnosis of primary CRC. Second, FCs of CRC patients who were treated in the three centers over the period were invited to participate in this study. Third, the FC of a CRC patient had to be a dedicated primary caregiver without receiving any monetary compensation, and be able to communicate with the interviewers. A list of eligible FCs was provided by the doctors. According to the list submitted by the doctors, we conducted a face-to-face survey of all the FCs on the list.

The structured questionnaire was administered *via* face-to-face questioning sessions in the three cancer centers. Eight post-graduate research students from the Harbin Medical University, trained as interviewers enrolled and were granted approval from the participating hospitals to carry out the study. All participants completed identical and validated questionnaires (SF-6D along with EQ-5D-3L) and provided sociodemographic information (8, 14). The research was granted approval by the Medical Ethics Committee of the Harbin Medical University (Daqing), and its ethical project identification code is 16HMUSCI032. Prior to the research, each subject was informed on the aim of the research, and they signed an informed consent form each.

**Figure 1:**
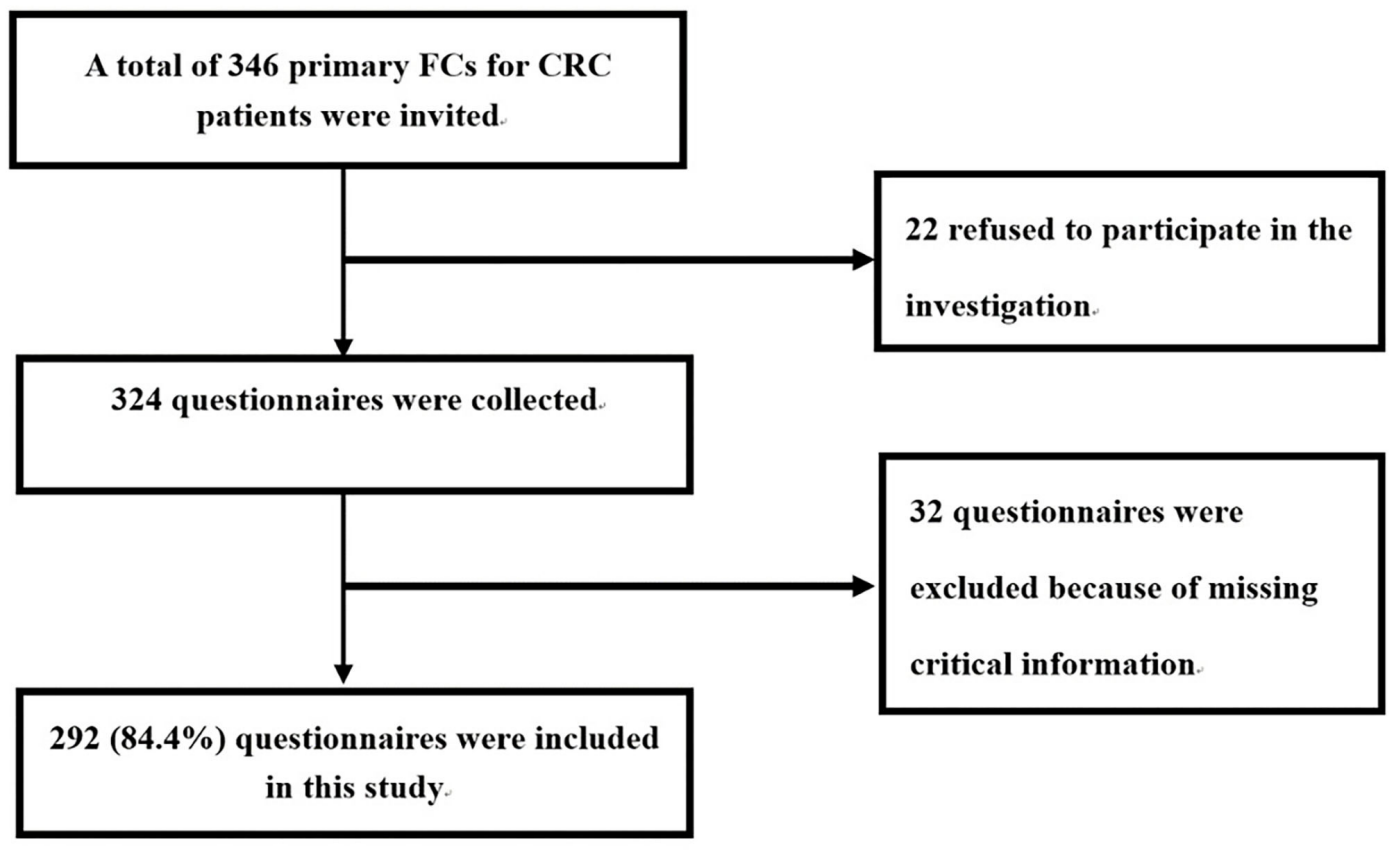
Flowchart of the questionnaire survey.

A total of 346 primary FCs for CRC patients were invited by the interviewers. Of the eligible participants, 22 refused to participate in the investigation after its purpose was explained to them. Therefore, a total of 324 questionnaires were collected. Eight trained interviewers checked the quality of the 324 questionnaires, and 32 questionnaires were excluded because of missing critical information in relation to the health utility data of the FCs and clinical characteristics of the patients. This resulted in a final sample size of 292 (84.4%) (Figure 1).

### Instruments

In the EQ-5D-3L, the participants were asked to rate the problems they experienced on a three-level scale (no problems, some problems, extreme problems) in relation to five health domains: mobility, self-care, usual activities, pain/discomfort, and anxiety/depression. The integration of the five dimensions for every subject was given a score index, as per the public preference. Besides, the subjects were asked to rate their general health on a 20-cm visual analog scale (EQ-VAS), which documents the self-rating health of the respondent as a score from 0 (worst health) to 100 (best health). Herein, we adopted the Chinese EQ-5D-3L value set to estimate the utility score for each FC of the CRC patient (15).

The SF-6D is a multidimensional health categorization system for defining health as abstracted from seven of the eight domains of health, defined by the SF-36v2 health survey. It adopts 11 questions of the SF-36v2 to describe the six domains consisting of social functioning, role limitation, physical functioning, mental health, vitality, and pain (16). Every domain has 4–6 levels of response, leading to an overall of 18,000 health conditions. A value set of Hong Kong was adopted to estimate the utility index for the SF-6D (16).

World Health Organization Quality of Life Questionnaire (WHOQOL-BREF) constitutes a well-established generic tool for assessing the HRQoL, and it has been verified in China (17). It consisted of 26 items, assessing physical health, social associations and environment, psychological health, and perceived overall life quality along with general health (18). Herein, we collected the overall quality of life item (WHOQOL-BREF-OQ) of FCs of the CRC patients. The WHOQOL-BREF-OQ was assessed with the following classes: “excellent,” “good,” “fair,” and “poor.”

### Statistical Analysis

The data were imported into the EpiData 3.1 and analyzed with the SPSS software, V. 20.0. All statistical tests were two-tailed and conducted at a 0.05 level of significance.

The distribution, median, agreement between the utility scores, and mean of the two instruments were compared. Ceiling effects was present when >15% of the FCs responded yielding the highest likely utility scores (19). The *t*-test was adopted to compare the within-subject differences of the two utility scores. Minimally important difference (MID) of utility score constitutes the smallest change in a patient-reported outcome that would result in a change of treatment. In our study, 0.074 and 0.041 were adopted according to the published data of MID for the EQ-5D-3L, as well as SF-6D (20). The interclass correlation coefficient (ICC) along with a Bland–Altman plot were adopted to explore the degree of agreement of the two utility scores. An ICC <0.4 depicts dismal agreement, 0.4–0.59 designates fair, 0.6–0.74 designates good, and 0.75–1 designates excellent agreement (21).

For the truth aspect, the convergent validity was compared with Spearman’s correlation between the two utility scores and the SF-6D scores, EQ-VAS, and the overall WHOQOL-BREF-OQ. In addition, we assessed the discriminatory capacities of the two health utility scores to differentiate between subjects with different health conditions. Study subjects with different health levels were categorized as per the WHOQOL-BREF-OQ. The ability to distinguish between WHOQOL-BREF-OQ “excellent” relative to “good,” “good” relative to “fair,” and “fair” relative to “poor” subgroups was computed using one-way ANOVA. Finally, known-groups validity was assessed using ANOVA. We computed the effect size (ES) according to the standardized mean difference documented by Cohen (22). The ES was divided into large (>0.8), moderate (0.5–0.8), or small (0.2–0.5). The ability of the two health utility scores to reveal the difference in the health condition of WHOQOL-BREF-OQ was assessed *via* the relative effective (RE). RE is computed based on the ratio of the square of the *t*-statistic of the EQ-5D-3L utility score to the *t*-statistic square of the SF-6D. An RE of more than 1 illustrates that EQ-5D-3L is more effective relative to the SF-6D in revealing the difference. When the RE is <1, the reverse is true (23).

## Results

Overall, 346 FCs of individuals with CRC were approached and 292 (84.4%) returned valid questionnaire for data analyses (age *M* = 45.8 years, SD = 12.1). Characteristics of FCs of all the sample are given in Table 1. Majority of the FCs were female (59.2%). The FCs exhibited an average experience of 4.2 months (SD = 9.8) of caring for the individuals with CRC, and they averagely spent 18.2 h (SD = 7.6)/day.

**Table 1:** Participant characteristics and Sociodemographic.

**Variable**	***N*** **(%)**	**Mean (SD)**
**Characteristics of family caregivers (FCs)**		
Gender		
Male	119 (40.8%)	
Female	173 (59.2%)	
Age		45.8 (12.1)
Ethnicity		
Han	285 (97.6%)	
Other	7 (2.4%)	
Duration of caregiving (Month)		4.2 (9.8)
Hours of caregiving per day (Hour)		18.2 (7.6)
Relationship to patient		
Child	128 (43.8%)	
Spouse and Parent	134 (45.9%)	
Other	30 (10.3%)	
Education		
No more than primary school	24 (8.2%)	
Middle or high school	161 (55.1%)	
University and above	107 (36.6%)	
Marital status		
Married	257 (88.0%)	
Other	35 (12.0%)	
WHO QOL-BREF-OQ		
Poor	31 (10.6%)	
Fair	83 (28.4%)	
Good	142 (48.6%)	
Excellent	36 (12.3%)	
EQ-5D-VAS		78.39 (14.14)
EQ-5D-utility		0.88 (0.11)
SF-6D-utility		0.86 (0.11)

**Figure 2:**
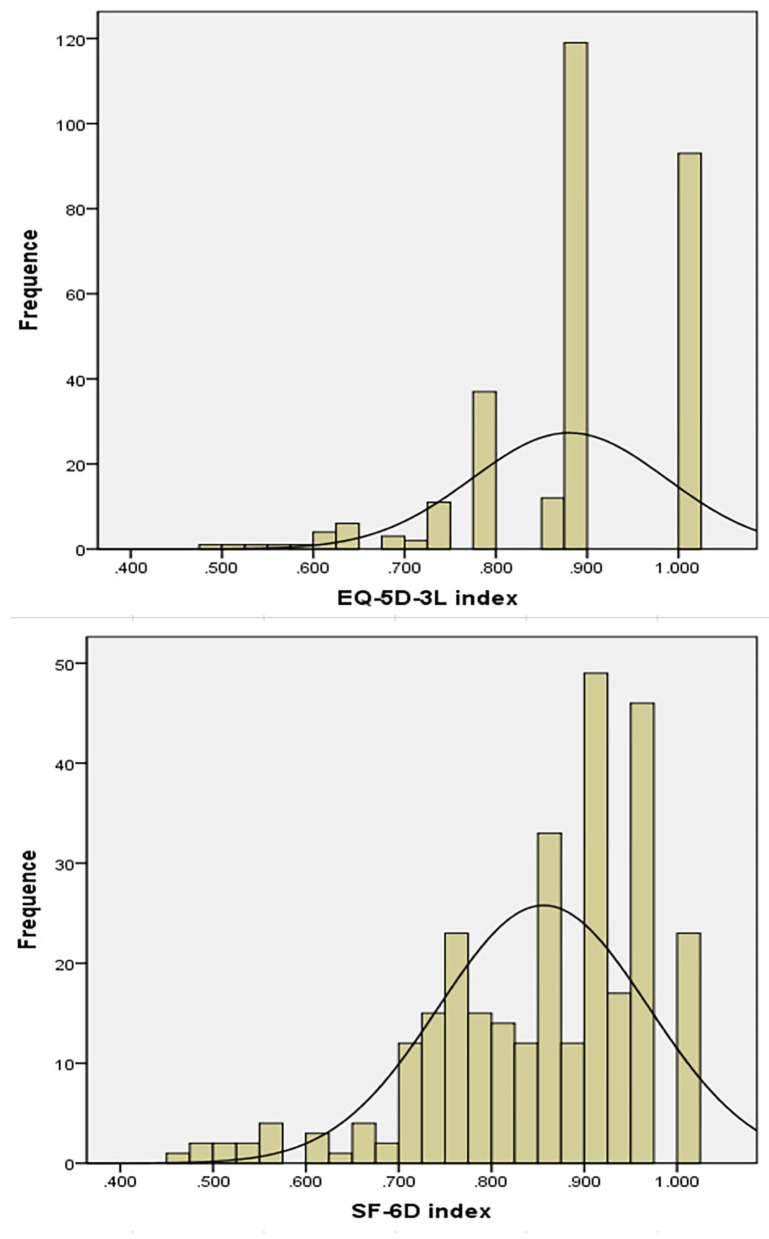
Score distribution of the EQ-5D-3L and SF-6D.

**Figure 3:**
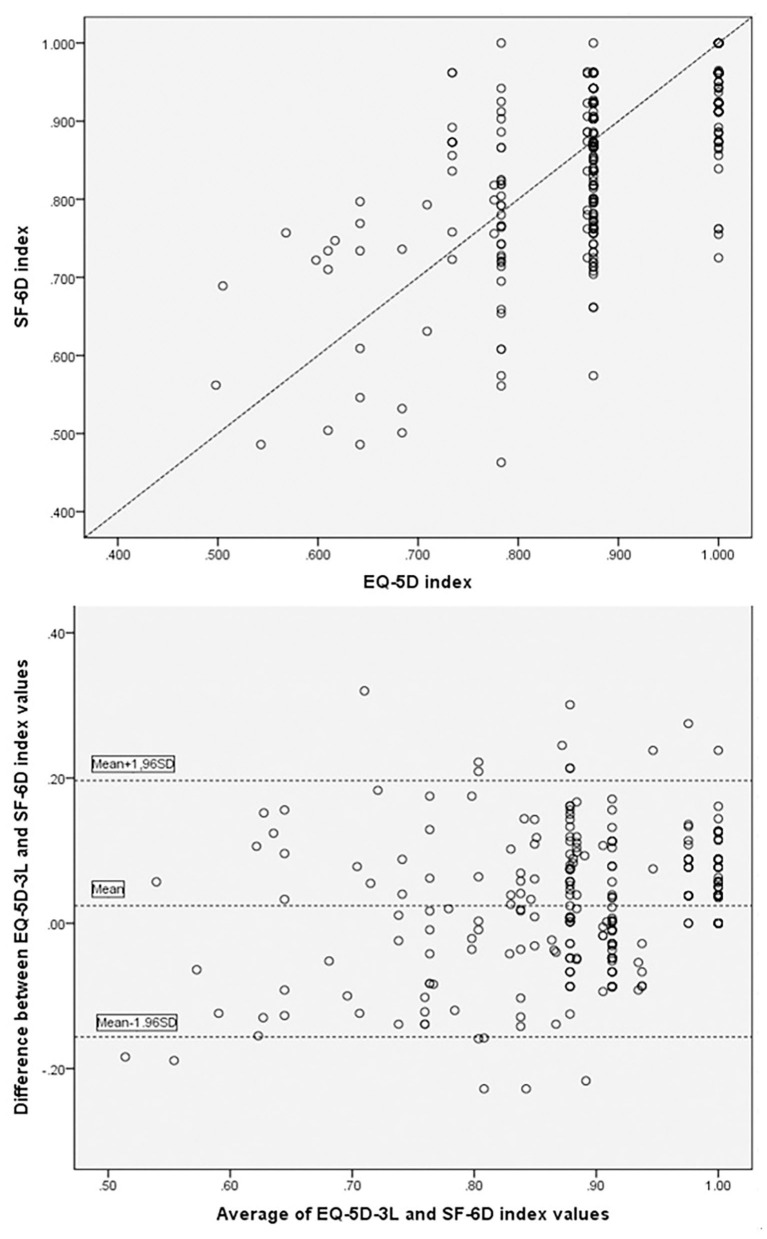
Paired utility scores for the entire population and Bland-Altman plot.

The score distribution for the EQ-5D-3L (skewness = 0.861, kurtosis = 0.874, *p* = 0.000) and SF-6D were not normal (skewness = −1.62, kurtosis = 1.051, *p* = 0.000; Figure 2). The two most frequently documented EQ-5D-3L distributions were 11122 (40.8%) and 11111 (31.8%), whilst the documented SF-6D distributions were distributed across all the states, none was documented *via* >11.0%. A ceiling effect was seen in the EQ-5D-3L, in which 20% of the subjects responded with the highest likely score. No ceiling effects were seen in the SF-6D. EQ-5D-3L, as well as the SF-6D mean utility scores were 0.88 (±0.11) and 0.86 (±0.11), (*p* = 0.000). The utility scores median of EQ-5D-3L and SF-6D were 0.875 (IQR 0.131) and 0.879 (IQR 0.162). A mean difference of 0.02 (±0.09) was seen between the two utility scores, which was lower relative to the MID of the EQ-5D-3L and the SF-6D. As is illustrated in Figure 3, the Pearson’s *r* between the two instruments was 0.622 (*p* < 0.01). Substantial agreement between the two utility scores was demonstrated with a good ICC (ICC = 0.637, average ICC = 0.778) for the entire population. In Figure 3 (Bland–Altman plots), a fraction of 94.2% of the differences lied within the 95% agreement limits (−0.156, 0.196), with more outlier differences dispersed above the high limit (3.4%) than below the low limit (2.4%), illustrating that the EQ-5D-3L index scores were slightly higher than the SF-6D ones.

For convergent validity, the Spearman’s R between the EQ-5D-3L and WHO QOL-BREF-OQ and EQ-VAS were moderate (0.446–0.457), and the associations between the SF-6D and WHO QOL-BREF-OQ and EQ-VAS were also moderate (0.463–0.483; Table 2). There were moderate correlations at the domain level between the two tools that assess a similar convergent. The Spearman’s *R* were 0.62 (*p* < 0.01) between EQ-5D-3L pain/discomfort and SF-6D pain, and 0.627 (*p* < 0.01) between EQ-5D-3L depression/anxiety and SF-6D mental health. The EQ-5D-3L mobility, usual activities, and self-care correlated weakly with the SF-6D physical functioning (*r* = 0.144–0.269), demonstrating that these domains assessed diverse aspects of HRQoL.

**Table 2:** Correlation of EQ-5D and SF-6D with WHO QOL-BREF-OQ and EQ-VAS.

	**SF-6D utility**	**WHO QOL-BREF-OQ**	**EQ-VAS**
EQ-5D utility	0.622^*^	0.457^*^	0.446^*^
SF-6D utility	–	0.463^*^	0.483^*^

* *p < 0.01*.

For discriminant potential, the EQ-5D-3L differentiated between subjects with “Poor” or “Fair” health condition, with strong ESs. The SF-6D differentiated with “Fair” relative to “Good,” “Good” relative to “Excellent” health condition with only moderate ES (Table 3). Given the difference of “Fair” from “Good” of the WHO QOL-BREF-OQ, the RE score was 1.089, illustrating that the EQ-5D-3L was as effective as the SF-6D in distinguishing these two health conditions. The RE was 1.694 for distinguishing “Poor” from “Fair” of the WHO QOL-BREF-OQ. This suggested that EQ-5D-3L was 69.4% more effective relative to SF-6D in determining subjects with “Poor” or “Fair” health condition. In addition, the RE was 0.464 for differentiating “Excellent” and “Good.” This indicated that the SF-6D was more effective relative to the EQ-5D-3L in determining subjects with “Excellent” or “Good” health condition.

**Table 3:** Discriminant capacity of EQ-5D and SF-6D utility scores.

		**Utility score**	p^*^	**Effect size**
EQ-5D	WHO QOL-BREF-OQ			
	Poor	0.747		
	Fair	0.855		0.973^**^
	Good	0.907		0.571^***^
	Excellent	0.949	<0.001	0.472^****^
SF-6D	WHO QOL-BREF-OQ			
	Poor	0.730		
	Fair	0.823		0.727
	Good	0.882		0.596
	Excellent	0.939	<0.001	0.655

* *One-way ANOVA*.

** *Comparison between Poor vs. Fair subgroup*.

*** *Comparison between Fair vs. Good subgroup*.

**** * Comparison between Good vs. Excellent subgroup*.

For known-groups validity (Table 4), both SF-6D and EQ-5D-3L showed sufficient ability to discriminate between the known groups. Participants who were females, spouse, and parent, older than 45 years, less education, married, longer duration of caregiving, tended to have lower mean utilities as measured by both SF-6D and EQ-5D-3L. Furthermore, the EQ-5D-3L was rather more efficient with respect to marital status (RE = 1.03) and duration of caregiving (RE = 1.24); and the SF-6D was rather more efficient with respect to gender (RE = 0.75), age (RE = 0.44), relationship to patient (RE = 0.67), and education (RE = 0.66).

**Table 4:** Known-groups validity and relative efficiency of the EQ-5D-3L and SF-6D.

**Variable**	***n*** **(%)**	**EQ-5D-3L**			**SF-6D**			RE^*^
		**Mean**	**SD**	***P***	**Mean**	**SD**	***P***	
**Characteristics FCs**								
Gender								
Male	119	0.905	0.089	0.001	0.887	0.099	<0.001	0.75
Female	173	0.863	0.114		0.835	0.117		
Age								
≤45	154	0.897	0.097	0.004	0.883	0.097	<0.001	0.44
>45	138	0.861	0.113		0.826	0.122		
Relationship to patient								
Child	128	0.889	0.079	<0.001	0.901	0.090	<0.001	0.67
Spouse and Parent	134	0.816	0.129		0.850	0.116		
Other	30	0.895	0.099		0.929	0.086		
Education								
No more than primary school	24	0.858	0.127	0.012	0.840	0.083	0.001	0.66
Middle or high school	161	0.868	0.108		0.837	0.124		
University and above	107	0.904	0.952		0.887	0.092		
Marital status								
Married	257	0.873	0.106	0.002	0.848	0.115	0.002	1.03
Other	35	0.932	0.095		0.911	0.078		
Duration of caregiving (Month)								
≤1	186	0.900	0.093	<0.001	0.876	0.099	<0.001	1.16
>1	106	0.845	0.118		0.822	0.127		
Hours of caregiving per day (Hour)								
≤12	110	0.893	0.096	0.122	0.868	0.107	0.164	1.24
>12	182	0.873	0.112		0.849	0.116		

* *Relative efficiency of one-way Anova F-statistics (F-statisticEQ-5D/F-statisticSD-6D)*.

## Discussion

Appropriate and valid utility index is the premise of cost-utility assessment. Hence, it is urgent to comprehend the efficiency of diverse indirect utility tools in diverse conditions. Herein, we provided and compared utility data of the SF-6D and the EQ-5D-3L with regard to distribution and agreement, convergent validity, discriminant validity, and known-groups validity in a cohort of the FCs of CRC patients recruited from three cancer centers located in the Heilongjiang province. The mean utility score of the EQ-5D-3L, at 0.88, was significantly higher than that of the SF-6D, 0.86 (*P* = 000). This is due to the percentage of reports on any health problem for the EQ-5D-3L being lower than that for the SF-6D. A previous study indicated that the EQ-5D-3L allows respondents with a slightly worsened health state to be reported as having a full health state (24). FCs of cancer patients are not cancer patients, and their health level will not be catastrophically affected. A previous study showed that significantly higher health utility scores for the EQ-5D-3L were found among the general population and rheumatoid arthritis patients (25). However, sometimes the score of SF-6D is higher than that of the EQ-5D. A Greek study showed that in individuals with clinical symptoms, the scores of SF-6D were predominantly higher than that of the EQ-5D-3L (26). A U.K. study showed that in patients with diseases such as chronic obstructive airway disease and irritable bowel syndrome, the mean score of SF-6D is higher than that of the EQ-5D (27). In this study, we found that the difference among the utility scores calculated by the two instruments was 0.02, which is lower relative to the smallest published MID value (20). Therefore, the difference in EQ-5D-3L scores from SF-6D scores was considered as clinically irrelevant.

Our research additionally indicated good agreement of ICC with utility scores generated *via* the two instruments. Similar findings have been documented in cohorts of pompe, mental health patients, HIV/AIDS patients, asthma patients, and patients with chronic obstructive pulmonary disease (28–31). A common conclusion in comparison investigations illustrates that health utilities of the EQ-5D-3L tend to be higher relative to those of the SF-6D in subclasses with better health, with the reverse being true in the poorer health subclasses (32). In our cohort, there were more positive values for the difference between the two instruments in the higher end of average score on a Bland–Altman plot. This means that systematic differences were seen in the mean difference of the utility scores of SF-6D and EQ-5D-3L with higher EQ-5D-3L scores at high mean utility scores. This is mainly because FCs of cancer patients generally report more problems in anxiety/depression domain. However, the EQ-5D-3L has only a three-level scale (no problems, moderate anxiety/depression, extreme anxiety/depression anxiety/depression). FCs with mild anxiety/depression may be underestimated as having no problems. This leads to the overestimation of the EQ-5D-3L at a high value compared with the SF-6D, and also leads to a ceiling effect. However, it was worth nothing that there is no difference between the two instruments in the lower end of average score in our study. This is because although the HRQoL of the FCs caring for cancer patients is lower relative to the general population (33, 34), the HRQoL of the FCs is higher relative to that reported in previous studies (35). For FCs caring for cancer patients, less problems were reported in mobility, self-care, as well as usual activities domains compared with other diseases (33).

A remarkable difference was observed in the EQ-5D-3L, while SF-6D exhibited no ceiling effect. This was consistent with the result of a previous study, in which the EQ-5D-3L exhibited a higher ceiling effect relative to the SF-6D (35). This is mainly because the number of response level of each EQ-5D-3L dimension is limited. The distribution for responses for the dimension “anxiety/depression” makes this problem especially evident for FCs. FCs (36.3% of the sample) can only report “no problems” on the anxiety/depression dimension, as the moderate/most level does not define their condition adequately.

In terms of “truth,” the efficiency of the EQ-5D-3a along with the SF-6D utility score were similar, with moderate association with the EQ-5D-3L VAS scores, as well as the WHO QOL-BREF-OQ. Distinguishing of EQ-5D-3L and SF-6D for different health states was assessed with ANOVA, ES, and RE. The utility scores differences across diverse health states were remarkably different (*p* = 0.000; ANOVA), which illustrates good discrimination. Furthermore, the EQ-5D-3L utilities performed efficiently in distinguishing subjects with poor and fair health conditions regarding ESs along with the RE scores, and the SF-6D utilities performed efficiently in distinguishing subjects with good and excellent health conditions. Known-groups validity was verified for both SF-6D and EQ-5D-3L. Nonetheless, the RE data showed a higher discriminatory efficiency of the SF-6D relative to the EQ-5D-3L version in the population defined by socio-demographic characteristics, except for marital status and duration of caregiving.

Utility scores are the pivotal composition of a cost-utility assessment. The tools employed to calculate the utility scores must be valid, and utilizing diverse tools should not affect the conclusions of an economic assessment. Data on the application of indirect HRQoL measures on the FCs caring for CRC patients is limited. Our research adds to the available literature on the comparison of the efficiency of EQ-5D-3L with SF-6D in FCs caring for CRC patients. Nonetheless, there has been little attention on the FCs in the cost-utility analysis of CRC interventions and screening (36, 37). We advocate for considering FCs of CRC patients, not only because the poor HRQoL of FCs may ultimately have a negative consequence on patient care outcomes, but also because considering the health utility scores of FCs into account will make the result of cost-utility analyses of the interventional activities more accurate.

There are several drawbacks to our study. First, this is a cross-sectional survey, the discrimination assessment is restricted to how the utilities distinguish diverse health conditions, rather than changing in condition with treatment over time. Second, we did not explore the reliability of these utility scores in FCs. Third, this research was conducted in a sample in China. Generalization of the current results to FCs in other countries should be done with caution. Finally, only FCs caring for CRC patients were studied; therefore, the findings may not be generalized to FCs caring for patients with other types of cancers.

## Conclusions

Despite these drawbacks, we have demonstrated in a cohort of FCs that the SF-6D performed slightly better in terms of convergent validity and discrimination of excellent health status, and improved known-groups validity efficiency than the EQ-5D-3L. Besides, SF-6D lacks ceiling effects. Utility scores of the SF-6D were lower relative to that of the EQ-5D-3L, but the difference may be clinically insignificant. However, the distinction may have a great effect on the conclusions of cost-utility evaluation. Further research is required to determine whether the EQ-5D-3L or the SF-6D is a better tool for cost-utility assessment in FCs.

## Data Availability Statement

The data of the current study are available from the corresponding authors upon reasonable request.

## Ethics Statement

The study was reviewed and approved by the Medical Ethics Committee of Harbin Medical University (Daqing), and its ethical project identification code is 16HMUSCI032. Before the study, each participant was informed about the purpose of the study and signed an informed consent form.

## Author Contributions

G-xL and XZ: acquisition of data, conceived the research idea, critical revision of the manuscript for important intellectual content, and conceived the research idea. YL, X-mZ, W-dH, L-rZ, and M-sW: conception and design and analysis and interpretation of data. C-yS: writing and drafting of the manuscript, analysis and interpretation of data, and statistical analysis. All authors have read and approved the final manuscript. All authors of this study have agreed to the publication of this manuscript.

## Funding

This work was funded by the National Natural Science Foundation of China (71673071) and National Key R&D Program of China (2017YFC1308700 and 2017YFC1308705).

## Conflict of Interest

The authors declare that the research was conducted in the absence of any commercial or financial relationships that could be construed as a potential conflict of interest.

## Publisher's Note

All claims expressed in this article are solely those of the authors and do not necessarily represent those of their affiliated organizations, or those of the publisher, the editors and the reviewers. Any product that may be evaluated in this article, or claim that may be made by its manufacturer, is not guaranteed or endorsed by the publisher.
